# A quantitative 3D intravital look at the juxtaglomerular renin-cell-niche reveals an individual intra/extraglomerular feedback system

**DOI:** 10.3389/fphys.2022.980787

**Published:** 2022-09-27

**Authors:** Patrick Arndt, Jan Sradnick, Hannah Kroeger, Stefan Holtzhausen, Friederike Kessel, Michael Gerlach, Vladimir Todorov, Christian Hugo

**Affiliations:** ^1^ Experimental Nephrology, Division of Nephrology, Department of Internal Medicine III, University Hospital Carl Gustav Carus, Dresden University of Technology, Dresden, Germany; ^2^ Institute of Machine Elements and Machine Design, Chair of Virtual Product Development, Dresden University of Technology, Dresden, Germany; ^3^ Core Facility Cellular Imaging, Experimental Center, Faculty of Medicine Carl Gustav Carus, Dresden University of Technology, Dresden, Germany

**Keywords:** intravital imaging, two-photon microscopy, renin cells, laser injury, glomerular injury, cell migration, ray tracing

## Abstract

The juxtaglomerular niche occupied by renin cells (RCN) plays an important role in glomerular repair but the precise temporal and spatial interrelations remain unclear. This study proposes the hypothesis of a local intra-extraglomerular regenerative feedback system and establishes a new quantifiable system for RCN responses in individual glomeruli *in vivo*. A strictly intraglomerular two-photon laser-induced injury model was established. Labeled renin cells (RC) in transgenic renin reporter mice were fate-traced in healthy and injured glomeruli over several days by intravital microscopy and quantified via new three-dimensional image processing algorithms based on ray tracing. RC in healthy glomeruli demonstrated dynamic extraglomerular protrusions. Upon intraglomerular injury the corresponding RCN first increased in volume and then increased in area of dynamic migration up to threefold compared to their RCN. RC started migration reaching the site of injury within 3 hours and acquired a mesangial cell phenotype without losing physical RCN-contact. During intraglomerular repair only the corresponding RCN responded via stimulated neogenesis, a process of *de novo* differentiation of RC to replenish the RCN. Repeated continuous intravital microscopy provides a state-of-the-art tool to prove and further study the local intraglomerular RCN repair feedback system in individual glomeruli *in vivo* in a quantifiable manner.

## Graphical Abstract

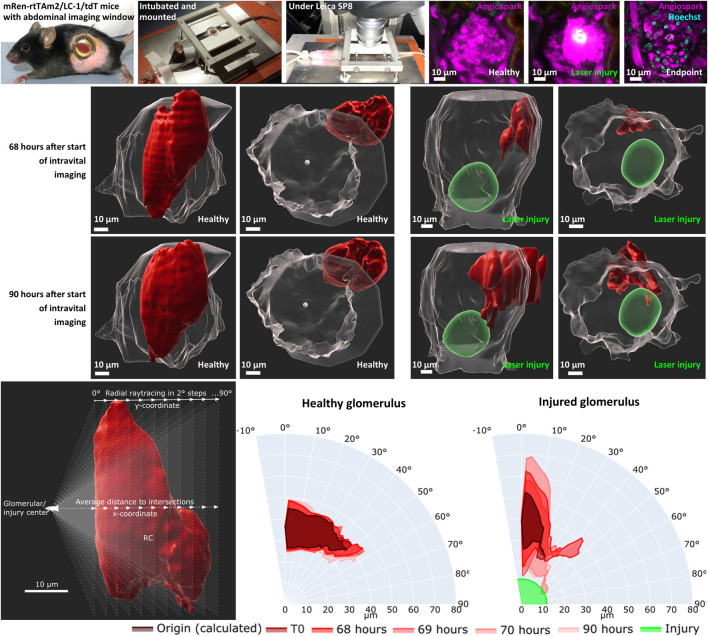

## Introduction

Specific renal progenitor cells occupy particular niches that have been identified to contribute to kidney regeneration and repair (Oliver et al., 2004; Starke et al., 2015; Andrianova et al., 2019). During nephrogenesis, stromal precursors differentiate into renal cell types that persist in the adult kidney (Sequeira Lopez and Gomez, 2011; Sequeira-Lopez and Gomez, 2021). Among them, renin cells (RC) reside in the juxtaglomerular apparatus (JGA) forming a renin cell niche (RCN) and function as pluripotent progenitors capable of migrating into the glomerulus after injury and acquiring a mesangial cell phenotype (Pippin et al., 2013; Pippin et al., 2015; Starke et al., 2015). In addition, juxtaglomerular RC are constantly replenished by neogenesis (*de novo* differentiation), a rare process of differentiation of a cell to join the renin lineage for the very first time under matured physiological conditions, that is markedly stimulated during response to injury (Hickmann et al., 2017; Steglich et al., 2020). While regulation of these complex repair processes is poorly understood, similar to the canonical tubulo-glomerular RC feedback mechanisms relating to salt-water homeostasis (Thurau and Schnermann, 1965), the authors hypothesized that these remarkable processes in adults, which resemble embryonic nephrogenesis to some extent, also underlie a local individual intraglomerular-extraglomerular feedback mechanism (Steglich et al., 2020). Understanding of this feedback mechanism and its mediators could open the door for new treatments for glomerular disease via stimulating endogenous renal repair mechanisms.

This hypothesis could be directly studied if a model system would be available, in which RC can be continuously visualized, individual glomeruli can be specifically injured and longitudinally imaged until an injury-directed repair process is completed and possibly quantified. In antibody mediated inflammatory glomerular injury models, systemic inflammation leads to injury and activation of a broad range of kidney cell types complicating the experimental study of the principle of cause and effect. In these models, RC-mediated repair processes occur only focally and variably up to 10 days following disease induction (Starke et al., 2015), making it impossible to study this hypothesis via intravital microscopy (Ruhnke et al., 2018).

Therefore, intravital imaging was applied in RC transgenic mice with an inducible cell type specific marker representing a state-of-the-art technique allowing the longitudinal study of complex glomerular and extraglomerular responses to injury on a cellular level (Schiessl et al., 2020; Desposito et al., 2021; Gyarmati et al., 2021). It enables spatially and temporally detailed visualization of the complex three-dimensional structure of various renal cell compartments under physiological and pathophysiological conditions (Hohne et al., 2013; Burford et al., 2014; Hickmann et al., 2017). This method can also be used to induce local injury/damage limited to very few cells by targeted laser irradiation in individual glomeruli and to observe processes longitudinally under controlled conditions (Kaverina et al., 2017; Schiessl et al., 2020). In this study, injury was chosen to be strictly intraglomerular and distant from the JGA. Three-dimensional object information could be obtained directly by virtual reconstruction and rendering, and used for novel quantification via ray tracing techniques. Ray tracing is an established method in microscopic and optical object characterization in which the path of light through pixels is traced in individual image planes (Kostenko et al., 2013; Khitrin et al., 2017). It finds broad application in generating images in computer graphics. Combining these novel techniques would extend the current knowledge in glomerular imaging (Hackl et al., 2013; Kaverina et al., 2017). Precise intravital overview imaging can enable the rediscovery of corresponding cleared tissue areas and complement intravital imaging with fixed immunohistology to detect renin cell differentiation to mesangial cells (Renier et al., 2014; Klingberg et al., 2017).

The combination of site-specific glomerular injury induction and longitudinal observation with intravital two-photon microscopy in transgenic mice provides a direct view of the regenerative process involving site-directed RC migration and its underlying mechanisms (Zhang et al., 2020). This model system was developed to test the hypothesis of an intraglomerular-extraglomerular RC feedback system, since characterization of individual glomerular responses to injury can only be done in an artificial but timely and spatially controlled model system without broad and undefined systemically mediated injury.

## Materials and methods

### Animal experiments

**FIGURE 1:**
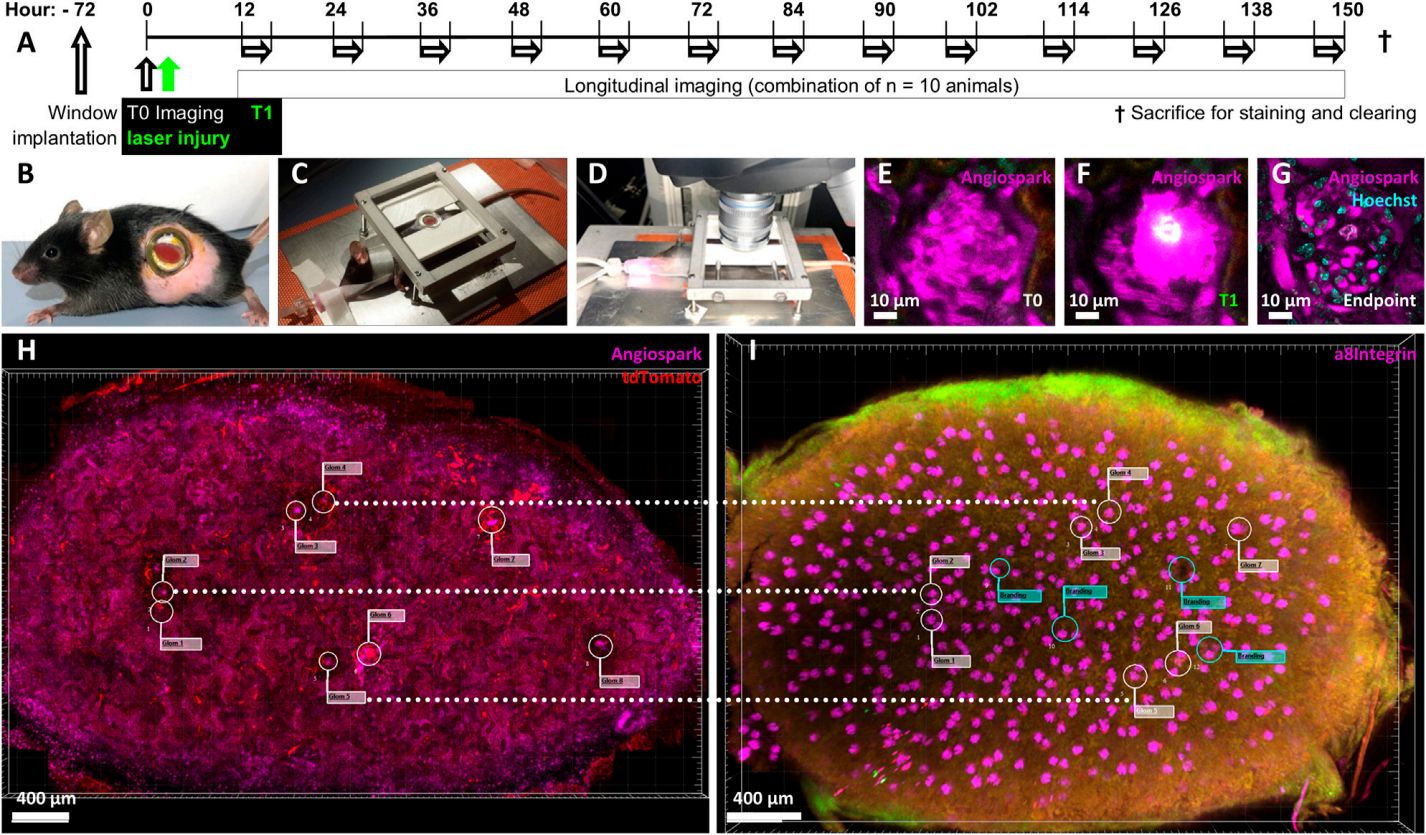
Experimental overview. Ten mRen-rtTAm2/LC-1/tdT mice underwent a 21-day pulse labelling induction period with 625 mg/kg Doxycycline and 10 mg/kg enalapril, followed by a 7-day washout period. **(A)** Timeline of experimental setup after pulse induction, washout and with abdominal imaging window implantation 72 h before baseline imaging (T0) followed by immediate laser injury irradiation (T1). Longitudinal imaging experiments from all mice were combined and overlapped to cover the entire time period according to the scheme. Each single mouse was imaged longitudinal for 3 h, always with at least 12 h recovery time and only to a maximum of 3 times. **(B)** Mouse with implanted abdominal imaging window in left flank and visible kidney (red) glued to the inside of the window. **(C)** Intubated, ventilated and anesthetized mouse on a heating plate fixed via the abdominal imaging window, **(D)** mounted for intravital two-photon microscopy. **(E)** Intravital imaged healthy glomerulus and its capillary structures made visible via **(I)** *v*. Angiospark 680 (magenta). **(F)** Laser-induced injury of the identical glomerulus with Angiospark 680 leakage and white autofluorescence of the laser-injured area. **(G)** Follow-up of the same laser-irradiated glomerulus 5 days after injury. The autofluorescent area is further visible in the still-functioning glomerulus revealed by Hoechst nuclear staining. **(H)** Kidney overview of glomeruli positions in three-dimensional intravital imaging data and **(I)** alignment to identical glomeruli after sacrifice, immunohistology staining and tissue clearing. Guiding laser brandings are marked with green flags in the overlay.

Eight-week-old female mRen-rtTAm2/LC-1/tdT mice underwent pulse induction via recombination with 625 mg/kg Doxycycline and 10 mg/kg enalapril for 21 days, followed by 7 days of washout without Doxycycline and enalapril (see Figure 1A) and were then used for imaging of healthy and laser-injured glomeruli (Ashworth et al., 2007; Steglich et al., 2019). Female mice are smaller compared to males and the nephrons are thus closer to the renal capsule (Schiessl et al., 2013). Due to the limited optical penetration depth of the laser, small mice are more suitable for intravital multiphoton microscopy. This approach also reduced (3R) the number of experimental animals. Constitutive mice double heterozygous for mRen and tdT/GFP (mRen-Cre-mT/mG) were used for neogenesis experiments (Hickmann et al., 2017). All mice were initially implanted with an abdominal imaging window (Figure 1B) as previously described (Schiessl et al., 2020). Under anesthetic tracheal intubation (isoflurane 1.5%, 0.8 O2 L/min) and after *i. v*. Injection of fluorescent agents Angiospark 680 (30 μL, undiluted, Perkin&Elmer) and Hoechst 33,342 (50 µL of 2 mg/ml water stock, Sigma-Aldrich), all mice were prepared for upright imaging on a heating plate with a custom-made abdominal imaging window holder (Figure 1C). For the laser injury characterization experiments only, Angiospark was exchanged for propidium iodide (50 µL of 2 mg/ml water stock, Sigma-Aldrich).

The animal study was reviewed and approved by TU Dresden and Landesdirektion Sachsen.

### Intravital two-photon laser scanning microscopy

Glomeruli were observed with an upright SP8 MP/OPO laser scanning microscope (Leica, Figure 1D) of the Core Facility Cellular Imaging (CFCI) for 3 hours on 3 days in different mice to cover seven consecutive days with intervals of at least 12 h each according to Figure 1A. Imaging was performed using a Leica HC PL IRAPO 40x/1.10 W (Wd = 0.65 mm) objective employing passive triggering by tracheal ventilation. Two-photon imaging was used with 860 nm/910 nm laser excitation, GFP detection at 525/50 nm, tdTomato detection at 617/81 nm and fared detection at 680/40 nm. Data was acquired with a pixel size of 0.362 µm × 0.362 µm and a Z-step size of 1 µm over a range of 120 µm. Glomeruli not deeper than 120 µm in the renal cortex (Figure 1E) were visualized in 10 mRen-rtTAm2/LC-1/tdT mice and in four mRen-Cre-mT/mG mice. The laser injury was induced by focusing 100% laser power for a maximum of 5 seconds at 48x zoom with a pixel size of 0.011 µm × 0.011 µm on one predetermined Z-plane. Individual intraglomerular Hoechst positive nuclei were carefully selected for each glomerular injury. Successful injury was characterized by strong local autofluorescence (Figure 1F). Injured glomeruli did not lose glomerular perfusion (Figure 1G). After the final imaging, a three-dimensional overview of the kidney area attached to the window was obtained (Figure 1H) and the examined kidney was removed.

### Kidney clearing and immunofluorescence staining

Intravitally imaged kidneys were perfused, harvested, the sections glued to the abdominal imaging window approx. 3 mm thick were cut and then immunolabeled with antibodies against α8Integrin (R&D systems, catalog-no. BAF4076) and tdTomato (anti-RFP, Rockland, catalog-no. 600-401–379) following the iDISCO protocol (Renier et al., 2014) and cleared with ethyl cinnamate (ECi) (Klingberg et al., 2017). Briefly, perfused kidney sections were treated in the following order: Paraformaldehyde fixation, methanol dehydration, H_2_O_2_ bleaching, H_2_O rehydration, permeabilization, blocking, antibody staining, methanol dehydration, embedding in agarose and finally ECi clearing. The cleared sample was microscoped entirely and reassessed without automation for the identical microscopic area captured prior to the final biopsy (Figure 1I). Selected glomeruli could be rediscovered by superimposed overviews, comparing landmarks such as laser brandings and identical orientations of glomeruli for detailed fluorescence imaging. Virtual green flags (Figure 1I) in the overlay marked laser brandings, guiding the manual rediscovery to completion.

### Data analysis

Three-dimensional image processing and analysis was performed with Imaris 9.7.2 (Bitplane AG). Here, surface models of the renin cells and injured areas were created via intensity thresholds across all compared and normalized time points. Glomerular borders were generated by defining object edges in multiple z planes and then automatically extrapolated. The glomerular centers were also automatically calculated by volume statistics of the glomerular borders. All surface models and images were corrected for spatial drift and aligned to one another in image stacks.

Novel automated three-dimensional image quantification was realized by the Marching Cubes algorithm. Image stacks were used to calculate a surface model described by triangles. The calculation of the directional thickness of the JGA in relation to the glomerular center in healthy glomeruli and to the injury center in laser-injured glomeruli was based on an adapted ray tracing method. A reference coordinate system was defined in these centers and used as the basis for further calculations. In this way, the point of origin $\vec{O}$ and the orientation were determined on the basis of the initial position of the objects relative to one another. The directional thickness was determined integrally by scanning individual rays, calculating the distance between the entry point and the exit point of the JGA. The tracing rays were calculated by a spherical coordinate system, with angles phi (φ) and theta (ϑ) defining the range of values of the tracing as given in Eq. 1, 2 (see Supplementary Material). The direction vector $\vec{X}$ of the ray tracing can be described by Supplementary Material Eq 3, with r=1 . With vector $\vec{X}$ , the ray tracing function $\vec{R\left(t\right)}$ is given in Eq 4 (see Supplementary Material). For each ray, the intersection points with the triangle mesh of the JGA were calculated. This calculation of the intersection points was carried out via a plane spanned by a triangle. This plane can be described with the normal $\vec{n}$ and the distance d to the origin as in Eq 5 (see Supplementary Material). By inserting Eq. 4, 5 and converting, the running variable t (Eq 6) was calculated (see Supplementary Material). Thus, Eq 7 (Supplementary Material) followed for the intersection point $\vec{Q}$ . This also defined the intersection point $\vec{Q}$ within the plane. For a triangle with the vertices $\vec{p_{1}}$ , $\vec{p_{2}}$ and $\vec{p_{3}}$ the intersection point $\vec{Q}$ can be determined by converting into so-called barycentric coordinates. Therefore, the intersection point $\vec{Q}$ lies within the triangle if Eqs, 8,9 are true (see Supplementary Material). The result is the first and last intersection point with the JGA for each ray in the specified value range of angles. For each of these angles, 36 measurements were performed at symmetrically distributed points and the mean value was calculated. The thickness of the JGA and distance to a given center in each plane was derived from this. A schematic representation is given in Figure 4A.

This novel image quantification method was applied to all intravitally imaged time points and the data was plotted in polar charts generated with the Python package plotly, as shown in two representations (Figures 4B,C). The amounts of the plotted areas between the maximal inner border and the minimal outer border of all time points (Eq 10, Supplementary Material) were calculated and determined the individual origin of RC in each glomerulus. Further absolute and relative quantification after ray tracing was performed by subtracting the plotted area of an inner triangle from the area of an outer triangle between rays in 2° steps (Eq 11, Supplementary Material).

Quantifiable image deviation was calculated with the Python packages opencv-python and numpy and used as a representative surrogate parameter for glomerular perfusion. In brief, images were converted in two-dimensional float32 values, from which pixel standard deviations were calculated.

### Statistics

Statistical data was calculated in GraphPad Prism 9.0. Linear regressions are indicated with corresponding *r* ^2^ factors. D’Agostino-Pearson omnibus normality tests confirmed Gaussian distributions. Data was compared either with Student’s t test, or with two-way ANOVA tests and Tukey’s multiple comparisons test calculated individual mean ± SD values. P < 0.05 indicates statistical significance.

### Study approval

All procedures were prospectively approved by the local authorities (TU Dresden and Landesdirektion Sachsen).

## Results

**FIGURE 2:**
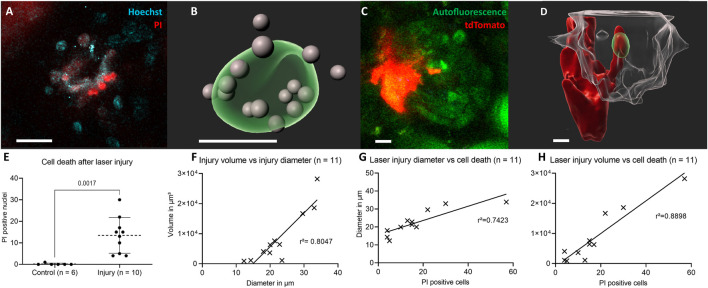
Characterization of the laser-induced injury model. **(A)** Two-dimensional slice view with Hoechst and PI stainings around the laser injury and **(B)** corresponding three-dimensional rendering of the autofluorescent area of a glomerular laser injury (green) with propidium iodide (PI) and Hoechst double positive cell nuclei in grey. **(C)** Two-dimensional slice view of renin cells (red) directly invading the laser injured area (green) and **(D)** corresponding three-dimensional rendered glomerulus (transparent-white). **(E)** Number of propidium iodide and Hoechst double positive nuclei 30 min following laser irradiation compared to control. Mean ± SD are indicated and compared with unpaired, two-tailed *t* test calculating *p* = 0.017 for statistical significance. **(F)** Positive linear correlation between laser injury volume and diameter. The geometric parameters were calculated with object statistics in Imaris 9.7.2 based on the autofluorescent area of the laser injury. The underlying geometric mean diameter amounted to 22.60 ± 7.05 µm and the volume to 8604 ± 8818 μm^3^ and were normally distributed in each case. **(G)** PI positive cells and laser injury diameter of autofluorescent area. **(H)** PI and laser injury volume of autofluorescent area. White scale bars represent 10 µm.

Figure 1 demonstrates the experimental setup, in which pulse labelled transgenic mice were baseline and then longitudinally repetitively imaged either without or with/after intraglomerular laser injury (for details see methods). Targeted use of laser irradiation established an inducible, selective and reproducible intraglomerular injury model of individual glomeruli (Figure 2). After laser irradiation, propidium iodide (PI) and Hoechst double positive dead cells appeared (Figures 2A,B,E). Injury diameter, volume and number of PI-positive cells correlated linearly (Figures 2F–H). After laser injury, site-directed migration and infiltration of RC into the injured area could be observed intravitally, as seen two-dimensionally in Figure 2C. The same set of data is presented three-dimensionally in Figure 2D with RC (red) entering the autofluorescent area (green) of laser injury, while still being connected to the RC within the JGA.

**FIGURE 3:**
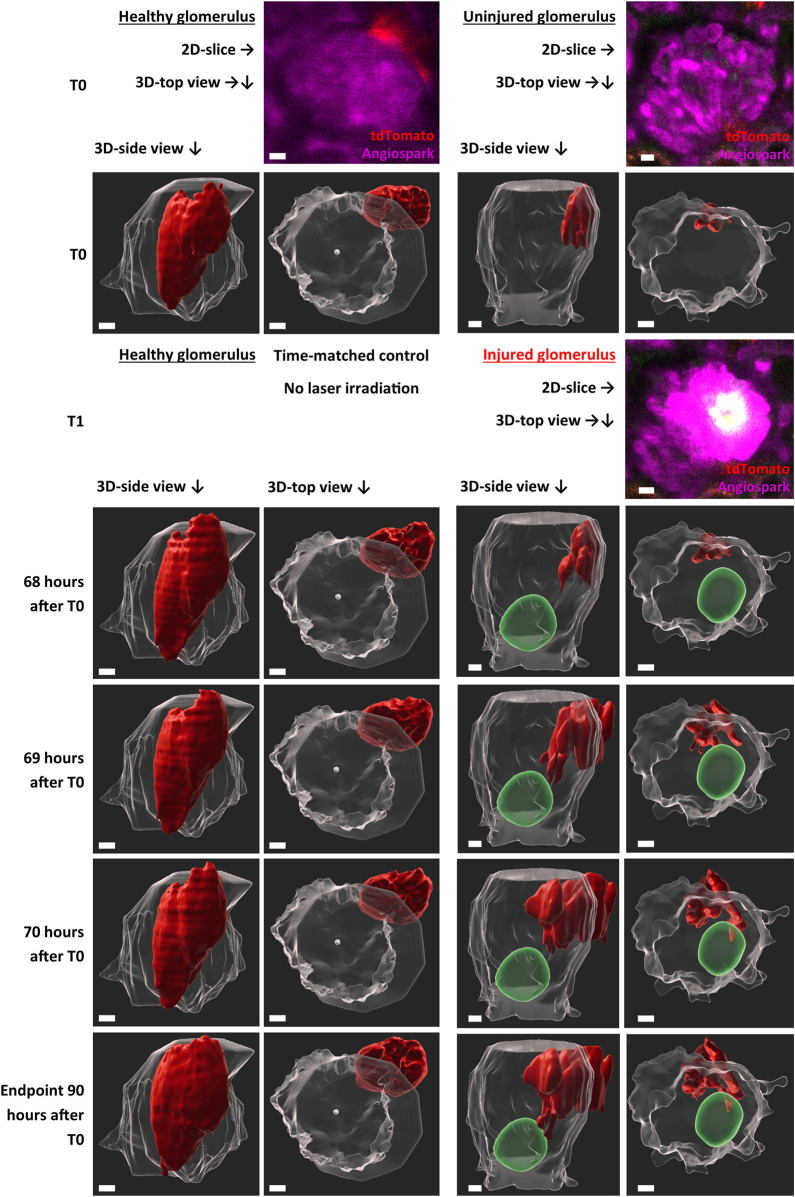
Representative intravital imaging data of physiological renin cell motion and pathophysiological migration. Z-stack imaging data of two time-matched glomeruli were rendered with Imaris 9.7.2 for the shown time points. T1 (laser irradiation) was applied in one glomerulus (right side). The individual migration in the injured glomerulus began after 68 h. Two three-dimensional-view points are given, side view of the glomeruli and top view along the *Z*-axis. TdTomato-labelled renin cells are presented in red. The autofluorescence of the laser injury was rendered in green. Glomerular border and center, automatic calculated with object statistics in Imaris 9.7.2 are marked in transparent white. White scale bars represent 10 µm.

TdTomato-labelled RC of the JGA in healthy glomeruli showed a constant flux of surface motion (representative healthy glomerulus in Supplement Video one and in Figure 3) over time. This active motion consisted of reversible protrusions appearing and disappearing within minutes (Supplementary Video S1) or over several hours and days as demonstrated in Figure 3 (T0—Endpoint).

With this established laser-induced injury model, the behavior of RC under pathophysiological conditions was analyzed intravitally (Figure 3). Targeted migration towards the area of glomerular injury was observed (representative injured glomerulus in Supplementary Video S2 and in Figure 3). Starting time points of RC migration varied between glomeruli, starting after 68 h in the representative injured glomerulus (Figure 3). At these time points protrusions from the juxtaglomerular RCN began to infiltrate the intraglomerular region. These migrating RC extended during 3 h towards the injury area, infiltrating the vicinity of the injured area without losing contact to the juxtaglomerular RCN.

**FIGURE 4:**
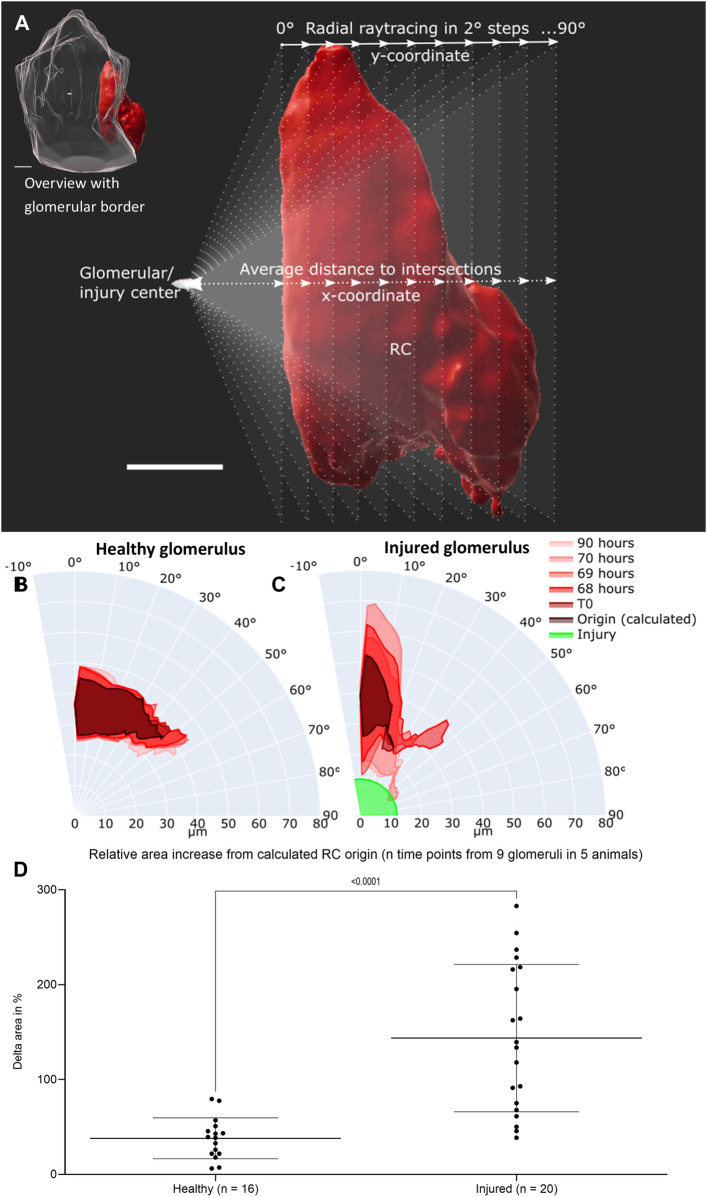
Intravital data (representative healthy and injured glomeruli) was quantified via average distance over view angles in 2-degree steps based on ray tracing. **(A)** Schematic representation of fan-like object quantification for healthy and injured glomeruli. White scale bars represent 10 µm. The origin of raytracing RC under physiological conditions were set to the center of the glomerular object and under pathophysiological conditions to the center of the autofluorescent laser injury. Resulting healthy RC motion and migration after injury are pictured in polar charts. **(B)** Quantification of the representative healthy glomerulus with the given time points from Figure 3. **(C)** Quantification of representative injured glomerulus with the given time points from Figure 3. The individual origin of RC in all glomeruli were calculated by the sum of the plotted area between the maximal inner border and the minimal outer border of all time points. **(D)** Increase in relative plotted area (see Supplementary Table S1) of n time points from the calculated RC origin from nine healthy and injured glomeruli from five different animals. Mean ± SD are indicated and were compared with unpaired, two-tailed *t* test calculating a highly significant statistical difference. White scale bars represent 10 µm.

Using a novel quantification technique (see scheme in Figure 4A), the motion of healthy RC and their migration after glomerular injury were further characterized with larger group sizes. The majority of RC in healthy glomeruli remained in a constant position over all time points, pictured exemplary in overlays in a polar chart in Figure 4B. Numerically, the physiological motion differs from T0 to up to 156% after 90 h (Supplementary Tables 1–4).

Under pathophysiological conditions after laser irradiation, RC from injured glomeruli first broadened significantly around their origin before converging on the laser-induced injury and further infiltrating the damaged area (representative injured glomerulus in Figure 4C). During these processes the RC area increased relatively in injured glomeruli from 100% (T1) to up to 350% (Supplementary Tables 5–8).

The calculated origin area of the juxtaglomerular RC was defined as the amount of the plotted area between the maximal inner border and the minimal outer border of all time points. This origin area from all analyzed glomeruli (healthy glomeruli I–IV and injured glomeruli I–V) amounted to an average of 76.71 ± 16.14% of RC from the first intravitally imaged time-point (100%) of the respective glomerulus. All underlying individual glomerular origin areas are listed in Supplementary Tables 1–9. A level of dynamic change in RC was calculated by comparing the increase in relative plotted area of the RC from all time points to the corresponding calculated origin area in both groups of glomeruli (Figure 4D). This data demonstrated significant higher delta areas of migrating RC in injured glomeruli than of RC in healthy glomeruli (Figure 4F).

**FIGURE 5:**
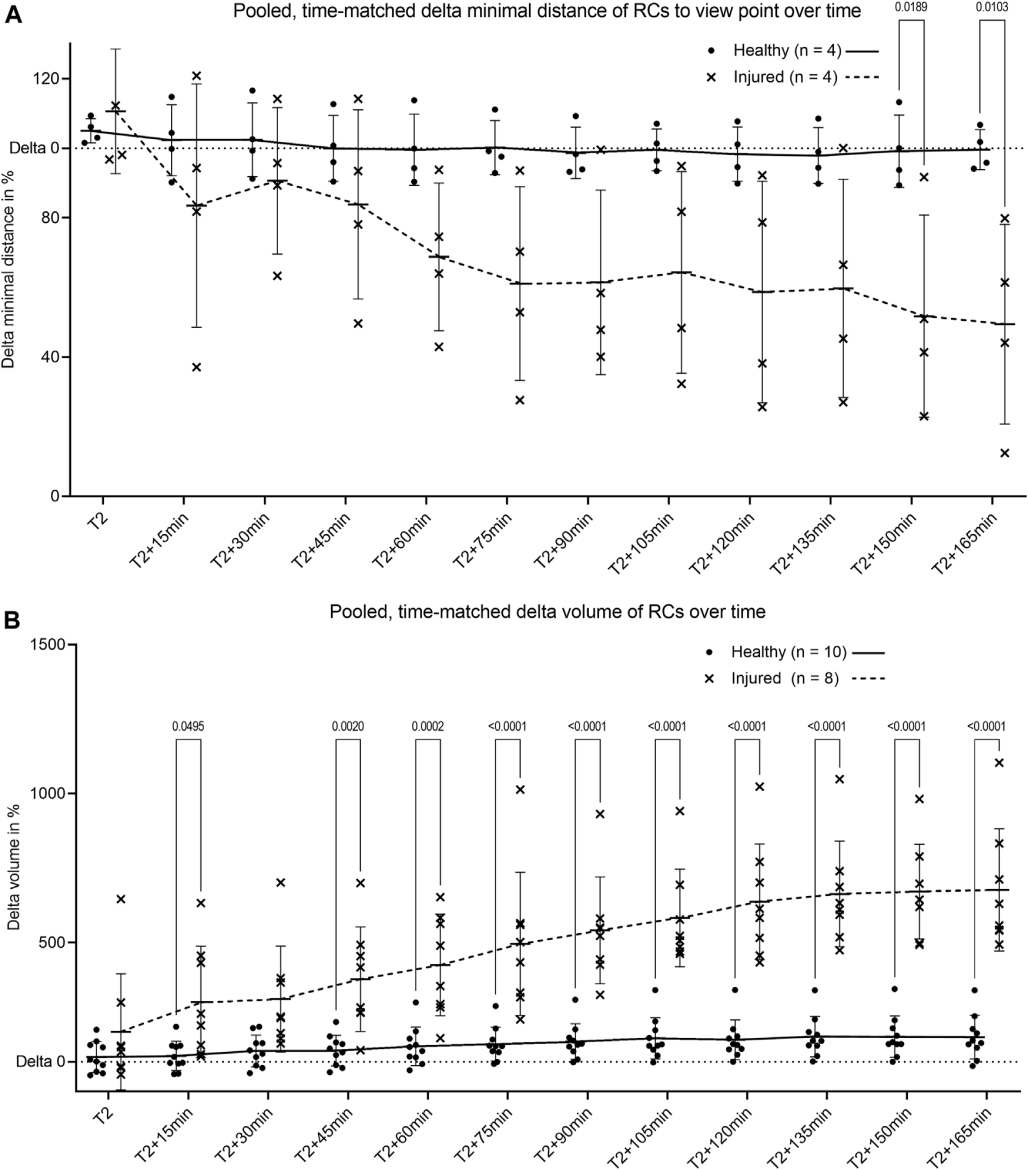
Pooled quantification of renin cell motion and migration. **(A)** Change of minimal distance between RC and their glomerular center for healthy glomeruli and between RC and the center of laser injury for injured glomeruli. The minimal distances were obtained by the novel automated three-dimensional image quantification based on ray tracing (*n* = 4 glomeruli per group each of four different animals). **(B)** Intravital change of renin cell volume over time in healthy glomeruli and in laser injured glomeruli. T2 defines begin of migration for injured glomeruli with matched time points from healthy controls. Renin cell volumes were calculated with object statistics in Imaris 9.7.2 based on the renderings of the tdTomato signal (n = 10 glomeruli per group each of 10 different animals). Healthy and injured data were compared with two-way ANOVA tests and Tukey’s multiple comparisons test calculated individual mean ± SD values. The corresponding mean values are connected by trend lines.

Comparison of pooled and time-matched RC migration based on the change of distance and volume revealed a decreased distance to the injured area (Figure 5A) and an increase in RC volume after laser-induced injury (Figure 5B). Longitudinal physiological surface motion of RC was also present, as indicated by non-significantly varying distances and RC volumes in healthy glomeruli in Figures 5A,B, respectively. RC migration processes reached the distant site of injury within the observation time of 3 hours.

**FIGURE 6:**
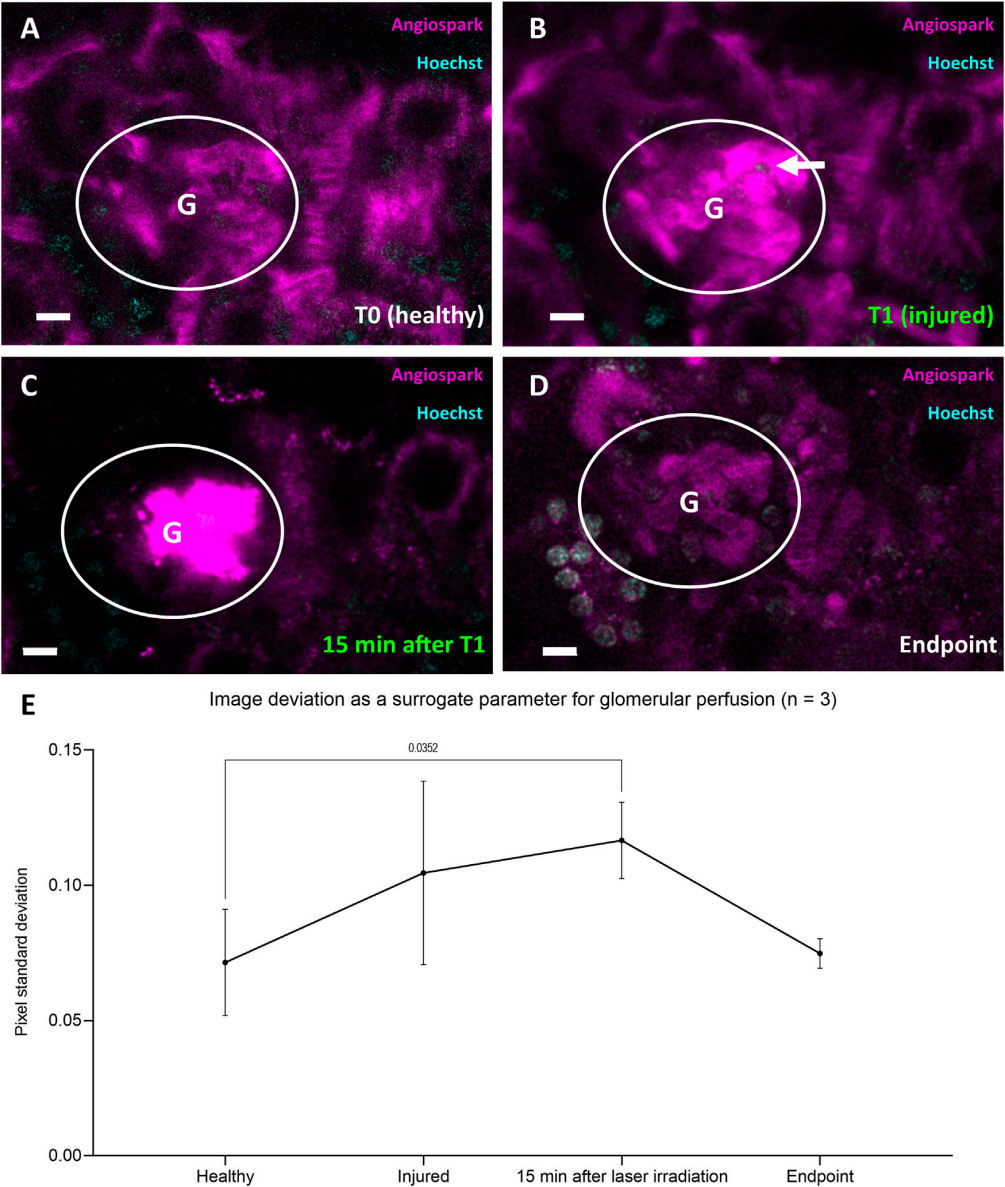
Representative data of glomerular perfusion before, during and after laser irradiation. **(A)** Representative glomerulus in white circle at four time-points healthy at T0 and **(B)** immediately after laser irradiation (white arrow) at T1. **(C)** Identical glomerulus 15 min after laser irradiation at T1 with visible Angiospark 680 leakage and **(D)** at experiment endpoint 96 h after T0. **(E)** Time-points **(A–D)** from three glomeruli of three different animals derived image deviation as a quantifiable surrogate parameter for glomerular perfusion. Image deviation data was compared with two-way ANOVA tests and Tukey’s multiple comparisons test calculated individual mean ± SD values. White scale bars represent 10 µm.

To demonstrate that laser injury did not lead to global glomerular death but rather to a RC-mediated repair reaction, repetitive longitudinal perfusion of glomeruli was monitored. Healthy perfused glomerular capillaries were visible through Angiospark 680 (magenta) during intravital imaging before laser irradiation (T0, Figure 6A). The Angiospark 680 intensity started to increase locally around the laser injury immediately after laser irradiation representative of capillary leakage (T1, Figure 6B) and increased to an overexposed intensity 15 min after laser irradiation (Figure 6C). Glomerular perfusion returned back to a physiological state after laser injury and subsequent RC migration intravitally visible at experiment endpoints, representative shown in Figure 6D. This process was quantitively assessed with the help of image deviation, a surrogate parameter for vascular perfusion in three glomeruli of three different animals (see Figure 6E). Healthy glomerular perfusion was defined as a homogeneously distributed Angiospark 680 filled vascular signal. The mean image deviation started at T0 under healthy conditions at 0.071 ± 0.019 and then increased to 0.105 ± 0.034 immediately after laser irradiation (T1) with a maximum of 0.117 ± 0.014 15 minutes after T1 (Figure 6E). The mean image deviation at the experiment endpoint returned to a similar initial value of 0.075 ± 0.005 as before laser irradiation (see also Figure 6E). A significant difference existed between healthy image deviation and image deviation 15 min after laser injury of *p* = 0.0352.

**FIGURE 7:**
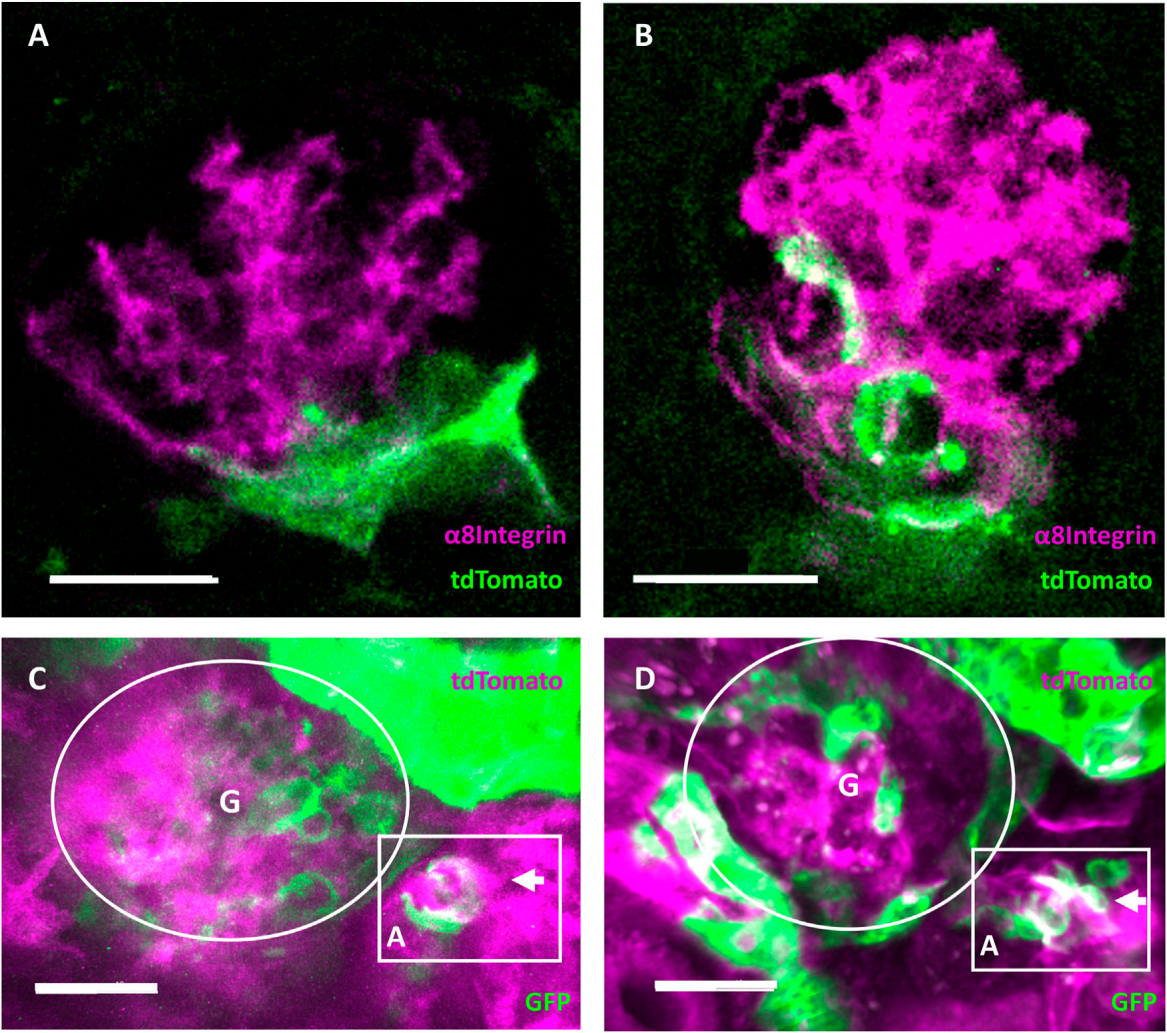
Evidence of regeneration and neogenesis during renin cell migration. **(A)** Healthy and **(B)** injured glomeruli from cleared mouse kidneys after intravital experiment endpoints. Complementary pseudocolors mark tdTomato immunohistological staining in green and α8Integrin in magenta. White areas indicate overlap in protein expression. The overlap of α8Integrin with tdTomato positive renin cell descendants after migration in injured glomeruli quantified to a mean of 32.71 ± 15.56% (*n* = 7). **(C)** RC neogenesis over **(D)** 6 days following renin cell migration in individually injured mRen-Cre-mT/mG mouse glomeruli demonstrated by single cell switch (arrows) from membrane-tdTomato-expressing non-RC (magenta) to membrane-GFP-expressing RC (green). Gradient tdTomato and GFP double positive signals are visible in white (magenta and green, respectively). Rectangular box highlights the neogenesis located at the JGA in three-dimensional intravital imaging data around the afferent arteriole **(A)** of one identical glomerulus **(G)**. A cluster of *de novo* differentiated RC (white and green cells in the rectangular box at d6) can be seen in the arteriole. White scale bars represent 20 µm.

Endpoint immunohistology with complementary pseudocolors further characterized RC migration with tdTomato positive RC (green) overlapping (white) with α8Integrin (magenta) in injured glomeruli but not in healthy glomeruli (Figures 7A,B, respectively).

Using constitutive transgenic mRenCre-mT/mG mice with GFP-expressing RC (green) and tdTomato-expressing non-RC (magenta), RC neogenesis important for maintaining the RCN can be identified over time, when *de novo* differentiated RC cluster can be observed via a gradient from tdTomato (non-RC) to GFP (RC marker, see Figures 7C,D). During repetitive intravital observations in uninjured glomeruli, this physiological process of RC neogenesis apparently occurred too infrequently to be documented. In contrast, after intraglomerular injury intravital observations of neogenesis only in the corresponding RCN were consistently detected by changing colors from magenta (non-RC) towards white (magenta and green for the first time) towards solely GFP-expressing RC (green) over time in the same cells. Representatively seen in Figures 7C,D, following laser injury mediated RC migration a *de novo* differentiated RC cluster was observed via a gradient from tdTomato (non-RC) to GFP (RC marker).

## Discussion

The presented novel quantifiable *in vivo* migration model system using longitudinal intravital microscopy combined with an intraglomerular laser-induced injury model allows new insights in the physiological and regenerative behavior and regulation of the RCN at the JGA. With the development of this model system, it is possible to describe and quantify fast and continuing alterations of RC protrusions under physiological conditions associated with its juxta/extraglomerular position. Applying intraglomerular injury to this model system in individual glomeruli showed that a locally acting feedback system gives notice of intraglomerular injury specifically to the associated JGA, leading to a timely (one to 3 days) and spatially coordinated repair response via directed migration of juxtaglomerular RC towards the site of injury. Hereby, the migrating RC switch their phenotype towards mesangial cells by expressing α8Integrin and during process completion continuously keep contact to the niche of origin. This repair response is also evident in the return to a physiological state of glomerular perfusion after laser injury and RC migration. The demonstrated glomerular leakage of Angiospark 680 filled vascular fluid represents a mechanical consequence of the laser irradiation and disappears a certain time after the onset of the repair response.

Besides the above described intra-/extraglomerular feedback repair mechanism, a second feedback system regulates specifically the maintenance of the RCN after intraglomerular RC migration. During the intraglomerular repair process by migrating RC, stimulation of their own replenishment via RC neogenesis was observed specifically in the corresponding RCN, a process in which nonRC switch their phenotype to a RC for the first time.

With this novel model in hand, understanding of the intra-/extraglomerular as well as RCN feedback mechanisms and its mediators could now open the door for new treatments for glomerular disease via stimulating/regulating these important endogenous renal repair mechanisms.

While laser-induced injury in individual glomeruli has been used before (Hackl et al., 2013; Zhang et al., 2020), the experimental setup was improved for reproducibility of injury areas. Treatment with enalapril was performed to induce the RC pulse labeling during induction, as previously published (Starke et al., 2015). Only an inducible reporter system ensures cell fate tracking and Ren promotor activity for a specific and short period of time, greatly reducing the risk of unwanted recombination, which is a limitation of constitutive reporter systems (Kaverina et al., 2017). The 7 day washout phase guaranteed the absence of experimental side effects because of enalapril’s short half-life of 35 h (Gomez et al., 1983). Using only 1.5% isoflurane as anesthesia administered via tracheal intubation excluded cardiovascular depression and provided a quick recovery of the animal after intravital imaging. Moreover, combining tracheal intubation with passive triggering controlled by the laser scanning microscope provided stable imaging without motion artifacts. In addition, the imaging technique had to be optimized and combined with novel computational algorithms to develop for the first time a quantifiable *in vivo* migration assay to study the hypothesis of a strictly locally regulated intraglomerular-extraglomerular feedback system for a RC-mediated response to injury in such a complex environment (Steglich et al., 2020). The use of the abdominal imaging windows markedly improves imaging conditions without pressure-induced restrictions of circulation (Schiessl et al., 2020). Intravitally imaged kidneys could be stained and cleared for translational immunohistology. Subsequent challenging but successful rediscovery of identical areas also demonstrated the mesangial phenotype switch of the migrating intraglomerular RC as a consistent finding as shown before in antibody induced systemic glomerulonephritis models (Starke et al., 2015).

Establishing an automated workflow of three-dimensional image quantification as presented here allows for unbiased and comparable analysis of cell behavior. Due to the calculation by the Marching Cubes algorithm, non-manifold, closed three-dimensional models were created, which were directly used for the semiautomated quantification of this complex three-dimensional longitudinal data. Representative shown image data was validated by statistically reliable comparisons. Cell motion and migration patterns can now be precisely calculated to detect spatial and temporal differences among glomeruli in further studies. A modest increase of relative area in healthy glomeruli over time is a limitation, but does not diminish the comparison to the results after laser-induced injury. The relative area, delta volume and delta minimal distance of RC between healthy and injured glomeruli are significant and, in several magnitudes, different. The introduction of thresholds for mathematical parameters will allow an easy distinction between fixed and dynamic cells. Interestingly, the start of visible RC recruitment occurred with some variability between one and at latest 3 days after injury, while the migrating RC frequently reached the intraglomerular site of injury within an observation time of 3 hours. The time point of RC recruitment may be influenced by the development of the injury/cell death itself, the corresponding functionality of the feedback players, of glomerular and/or tubular perfusion, or the (necro)inflammatory response (Sarhan et al., 2018). Once the feedback network and RC activation in individual glomeruli/JGA has been established, a quite uniform rapid migration of RC from the RCN towards the intraglomerular site of injury takes places with little variation in cellular speed.

This quantitative model system of the migratory response of RC after site-directed injury can now be used for comparative studies in different transgenic mouse strains or established therapies such as ACE-I, ARBs, SGLT-2 inhibitors, corticosteroids or may serve to find novel therapies.

Starting from the introduced hypothesis of a feedback system, several mechanisms may be relevant to inform the JGA about intraglomerular injury (Thurau and Schnermann, 1965). Either a chemoattractive gradient within the glomerulus towards the JGA, a cell junction mediated interconnective signaling mechanism, or an intraglomerular release of (death) molecules into Bowman space with a following interaction with the macula densa mimicking a real glomerular-tubulo-extraglomerular feedback mechanism may regulate the RCN and its responses. The model system presented here can be combined with new sophisticated technologies such as two-photon guided glomerular/tubular micropuncture and liquid chromatography/mass spectrometry of nanoliter range samples from recovered filtrates from Bowman space and segments of the nephron to answer the above questions (Matsushita et al., 2018). Subsequent studies utilizing the pluripotent (Pippin et al., 2015) potential of RC or further controlled intraglomerular (micro-)injection experiments (Ikeda et al., 2017; Saxena et al., 2021) will extend the presented research of RC-mediated repair and RCN replenishment regulation via neogenesis. In addition, the translational potential of this methodology remains high, as it can be applied to many image analysis questions.

In conclusion, repeated continuous intravital microscopy provides a state-of-the-art tool to prove and further study the local intraglomerular injury - extraglomerular RCN repair feedback system in individual glomeruli *in vivo* in a quantifiable manner. This model system may open the door for developing new treatments for glomerular disease via stimulating/regulating this important, endogenous renal repair mechanism.

## Data Availability

The original contributions presented in the study are included in the article/Supplementary Material further inquiries can be directed to the corresponding author.
