# Supplementary material for: A quantitative 3D intravital look at the juxtaglomerular renin-cell-niche reveals an individual intra/extraglomerular feedback system
Source: Front Physiol. 2022 Sep 27;13:980787. doi: 10.3389/fphys.2022.980787 (PMC9550881; doi:10.3389/fphys.2022.980787)
Supplement: Supplementary file 1 [file DataSheet1.PDF]

Supplementary Material

1 Supplementary Equations

1.1 Equation 1: Angle phi definition

$$\varphi = [0° \ldots 180°]$$

1.2 Equation 2: Angle theta definition

$$\vartheta = [-90° \ldots 90°] \; with \; step \; size \; 2°$$

1.3 Equation 3: Ray tracing direction vector

$$\vec{X} = \begin{matrix} r \cdot \sin\varphi \cdot \cos\vartheta \\ r \cdot \sin\varphi \cdot \sin\vartheta \\ r \cdot \cos\varphi \end{matrix}$$

1.4 Equation 4: Ray tracing function

$$\overrightarrow{R(t)} = \vec{O} + t \cdot \vec{X}$$

1.5 Equation 5: Plane calculation

$$\vec{x} \cdot \vec{n} = d$$

1.6 Equation 6: Variable t calculation with Equation 4 and Equation 5

$$t = \frac{\left(d - \vec{O} \cdot \vec{n}\right)}{\vec{X} \cdot \vec{n}} \; with \; \vec{X} \cdot \vec{n} \neq 0$$

1.7 Equation 7: Intersection point calculation

$$\vec{Q} = \vec{O} + \frac{\left(d - \vec{O} \cdot \vec{n}\right)}{\vec{X} \cdot \vec{n}} \cdot \vec{X}$$

1.8 Equation 8: True condition for intersection points

$$\vec{Q} = \overrightarrow{p_1} + \beta \cdot (\overrightarrow{p_2} - \overrightarrow{p_1}) + \gamma \cdot (\overrightarrow{p_3} - \overrightarrow{p_1})$$

1.9 Equation 9: True condition for triangles in barycentric coordinates

$$\beta \geq 0; \; \gamma \geq 0 \; and \; 1 - \beta - \gamma \geq 0$$

1.10 Equation 10: Plotted RC origin area calculation

$$\sum_{1,\vartheta=0°}^{90°} \max_{inner\ distance}(x_{1,\vartheta}) \bigwedge \sum_{2,\vartheta=0°}^{90°} \min_{outer\ distance}(x_{2,\vartheta})$$

1.11 Equation 11: Triangle area subtraction

$$\sum_{x,\vartheta=0°}^{90°} A_{x,o(outer\ triangle)} - A_{x,i(inner\ triangle)} = \sum_{x,\vartheta=0°}^{90°} \frac{1}{2} \cdot b_{x,o} \cdot c_{x,o} \cdot \sin 2° - \frac{1}{2} \cdot b_{x,i} \cdot c_{x,i} \cdot \sin 2°$$

2 Supplementary Videos

Supplement Video 1: https://caruscloud.uniklinikum-dresden.de/index.php/s/NMPmKpamcHmf47m

Supplement Video 2: https://caruscloud.uniklinikum-dresden.de/index.php/s/M6g8P4n9xEYMaQ8

3 Supplementary Tables

3.1 Table 1: Healthy glomerulus I

Time point	Plotted area in µm²	Relative area in %
Origin (calculated)	9226	95.45
T0	9666	100
68 hours after T0	12934	133.82
69 hours after T0	13411	138.75
70 hours after T0	13366	138.29
Endpoint	13627	140.98

3.2 Table 2: Healthy glomerulus II

Time point	Plotted area in µm²	Relative area in %
Origin (calculated)	6691	76.69
T0	8725	100
67 hours after T0	8252	94.58
67.5 hours after T0	8588	98.44
68 hours after T0	8955	102.64
Endpoint	10124	116.04

3.3 Table 3: Healthy glomerulus III

Time point	Plotted area in µm²	Relative area in %
Origin (calculated)	5940	77.80
T0	7635	100
0.5 hours after T0	7605	99.62
1 hours after T0	6488	84.99
1.5 hours after T0	6413	84.00
Endpoint	8438	110.53

3.4 Table 4: Healthy glomerulus IV

Time point	Plotted area in µm²	Relative area in %
Origin (calculated)	4392	76.87
T0	5714	100
41 hours after T0	7298	127.73
42.5 hours after T0	7649	133.88
44 hours after T0	8824	154.44
Endpoint	8932	156.33

3.5 Table 5: Injured glomerulus I: Laser irradiation at T1, start of migration at T2 = 68 hours

Time point	Plotted area in µm²	Relative area in %
Origin (calculated)	3051	66.85
T1	4564	100
T2	9150	200.49
T2 + 1 hour	10465	229.31
T2 + 2 hours	13861	303.73
T2 + 3 hours	15964	349.79

3.6 Table 6: Injured glomerulus II: Laser irradiation at T1, start of migration at T2 = 22 hours

Time point	Plotted area in µm²	Relative area in %
Origin (calculated)	4391	89.15
T1	4925	100
T2	12479	253.4
T2 + 1 hour	10201	207.12
T2 + 2 hours	15029	305.16
T2 + 3 hours	15151	307.63

3.7 Table 7: Injured glomerulus III: Laser irradiation at T1, start of migration at T2 = 34 hours

Time point	Plotted area in µm²	Relative area in %
Origin (calculated)	4090	38.14
T1	10726	100
T2	11363	105.94
T2 + 1 hour	9462	88.21
T2 + 2 hours	14046	130.95
T2 + 3 hours	8227	76.7

3.8 Table 8: Injured glomerulus IV: Laser irradiation at T1, start of migration at T2 = 72 hours

Time point	Plotted area in µm²	Relative area in %
Origin (calculated)	4626	76.83
T1	6021	100
T2	13023	216.28
T2 + 1 hour	19949	331.29
T2 + 2 hours	16395	272.28
T2 + 3 hours	18392	305.45

3.9 Table 9: Injured glomerulus V: Laser irradiation at T1, start of migration at T2 = 67 hours

Time point	Plotted area in µm²	Relative area in %
Origin (calculated)	5730	92.58
T1	6189	100
T2	8551	138.18
T2 + 1 hour	9518	153.8
T2 + 2 hours	10377	167.68
T2 + 3 hours	11373	183.78
